# Measuring Health-Related Quality of Life in Amyotrophic Lateral Sclerosis: A Systematic Review and Conceptual Framework

**DOI:** 10.1212/WNL.0000000000209549

**Published:** 2024-06-13

**Authors:** Emily McDool, Jill Carlton, Philip A. Powell, Elizabeth Coates, Liam Knox, Emily Mayberry, Niamh Appleby, Alys W. Griffiths, Esther Hobson, Christopher J. McDermott

**Affiliations:** From the Sheffield Centre for Health and Related Research (SCHARR) (E. McDool, J.C., P.A.P., E.C.), and Sheffield Institute for Translational Neuroscience (SITraN) (L.K., E. Mayberry, N.A., A.W.G., E.H., C.J.M.), University of Sheffield, Sheffield, United Kingdom.

## Abstract

**Background and Objectives:**

The assessment of health-related quality of life (HRQoL) in patients with amyotrophic lateral sclerosis (ALS) is heterogeneous and inconsistent. The objectives of this study were (1) to develop a comprehensive conceptual framework of HRQoL in ALS and (2) map the content of existing patient-reported outcome measures (PROMs) used in ALS to this novel framework.

**Methods:**

Our model of HRQoL in ALS (Health-related Quality of life in Amyotrophic Lateral Sclerosis, QuALS) was developed from a systematic literature review and consultative input from key stakeholders (patients, carers, and health care professionals). Five electronic databases were searched in April 2022. Primary studies of any design that assessed HRQoL in ALS by using a multi-item PROM and/or qualitative methods were identified. Using an a priori framework, HRQoL themes were extracted and iteratively modified from the content of each PROM and qualitative study quotations identified in the literature. The conceptual framework was ratified by stakeholders with lived experience and clinical experts. The QuALS framework was used to map the content of identified PROMs and qualitative studies based on thematic coverage.

**Results:**

QuALS covers 3 high-level domains of HRQoL (physical, psychological, and social functioning) and consists of 7 themes (Activities; Physical Health; Autonomy; Cognition; Feelings and Emotions; Self-identity; Relationships), characterized by 42 subthemes. Of 8,220 studies identified, 274 were included in the review that informed QuALS. In these studies, 111 PROMs were used to assess at least 1 aspect of HRQoL, and 11 studies used qualitative methods. Of the 3 high-level domains, physical functioning was the most commonly assessed, particularly within ALS-specific PROMs where almost one-quarter of PROMs exclusively assessed physical functioning. None of the PROMs or qualitative studies identified assessed all aspects of HRQoL in the QuALS framework.

**Discussion:**

This study presents a new comprehensive conceptual framework of HRQoL in ALS (QuALS), informed by a robust systematic review of existing literature and stakeholder input, incorporating lived experience. QuALS provides a valuable resource for researchers and clinicians interested in taking a holistic approach to assessing and understanding the full impact of ALS on HRQoL and how this may be affected by treatments.

## Introduction

Amyotrophic lateral sclerosis (ALS) is a rare life-limiting neurodegenerative disease that has a continual effect on patients' health-related quality of life (HRQoL), including physical, psychological, and social functioning.^1,2^ The impact of ALS on HRQoL may change with disease progression and/or in response to treatments.^1,3^ Recommendations relating to treatment and care in ALS focus on symptom management and alleviation, and the maintenance of HRQoL, as signaled in guidance from the National Institute for Health and Care Excellence and the American Academy of Neurology.^4,5^

HRQoL is a multidimensional concept that includes the physical, psychological, and social functioning associated with an illness or its treatment.^6^ HRQoL may be assessed qualitatively through interviews or focus groups with patients and/or their carers, or quantitatively using standardized questionnaires, commonly known as patient-reported outcome measures (PROMs). PROMs provide a subjective assessment of health outcomes (e.g., HRQoL, symptom burden) from an individual or through proxy reporting (e.g., informal carer, family member, clinician). New PROMs have recently been developed, designed to assess HRQoL (or a specific aspect of HRQoL) in ALS, including the preference-based ALS HRQoL scale^7^ and Rasch-Built Overall Amyotrophic Lateral Sclerosis Disability Scale.^8^ Across published studies, different approaches have been taken to assess HRQoL in people with ALS (pwALS). Frequently, studies focus upon a particular symptom (e.g., pain)^9^ or aspect of HRQoL (e.g., physical functioning) as opposed to taking a holistic approach^10^ and may use PROMs or qualitative methods that focus on a particular aspect of HRQoL. Generic PROMs (designed to be applicable across health conditions) have also been used^11^; however, they potentially fail to capture all aspects of HRQoL that are affected by, or are relevant to pwALS.^12^ In addition, there is a lack of consensus around which PROMs are most appropriate for use in ALS populations, in terms of their performance and coverage.^13,14^ A comprehensive model of HRQoL in ALS, based on what matters to people living with the condition, is needed.

The aim of this study was to develop a new comprehensive conceptual framework of Health-related Quality of life in Amyotrophic Lateral Sclerosis (QuALS), based on a robust systematic review of existing literature, with stakeholder input. The study had 4 objectives. First, to identify which PROMs have been used to measure the impact of ALS upon an individual's HRQoL (or at least 1 aspect of it); second, to develop an initial conceptual framework of HRQoL for ALS, informed by the HRQoL themes from the content of each of the identified PROMs and qualitative studies; third, to incorporate stakeholder feedback to ratify the QuALS framework; and finally, to map the content of identified PROMs onto the QuALS framework to produce a resource for researchers, clinicians, and other stakeholders to help identify the most appropriate instruments when wanting to measure HRQoL.

## Methods

To develop the QuALS framework, a multistage process was undertaken whereby an initial systematic review was conducted before stakeholder engagement (see eFigure 1). The aim of the systematic review was to identify studies that used qualitative methods and quantitative instruments (PROMs) to assess (an aspect of) HRQoL in pwALS, and to extract HRQoL themes from qualitative quotations and/or PROM content to inform the development of the initial conceptual framework.

The review protocol was registered with the International Prospective Register of Systematic Reviews (registration no: CRD42022323993).^15^ Systematic searches of MEDLINE (via Ovid), Embase (via Ovid), Psycinfo (via Ovid), CINAHL, and The Cochrane Library were conducted on April 27, 2022 to identify the literature and evidence on the PROMs used to assess HRQoL in ALS. No restrictions on language or date were applied to the searches. The search strategy was developed by an information specialist and consisted of all relevant subject headings and free text terms for ALS and motor neuron disease (MND). A customized filter of terms to represent HRQoL instruments was applied based on one developed in a previously published systematic review.^16^ eAppendix 1 (eTable 1) details the search strategy.

The screening process involved 6 paired reviewers (with records organized alphabetically and split into thirds across pairs), such that each pair included 1 reviewer with expertise in PROMs (E. McDool, J.C., P.A.P.) and 1 with expertise in ALS (E.C., L.K., E. Mayberry). Title and abstract and full text review stages were conducted, assessing records for inclusion against an a priori inclusion and eligibility criteria (Table 1). To improve the manageability of the review and focus on the contemporary measurement of HRQoL, inclusion was restricted to studies published post-2017. All titles and abstracts and full texts were dual reviewed and where disagreement within a reviewer pair occurred, a third reviewer ratified the inclusion decision. Studies selected for inclusion were read in full and dual data extraction was carried out within reviewer pairs. Data were extracted on the study title, author and publication year, and whether the study included any qualitative data, and/or which HRQoL PROM(s) (including version) were used. Copies of each PROM identified in the full text extraction process were obtained and reviewed by 3 reviewers collectively to assess whether the PROM met the predetermined inclusion criteria (Table 1). Data were extracted by 1 of 3 reviewers (E. McDool, J.C., P.A.P.) from each PROM on the name, version, recall period, response options (including type [i.e., frequency, severity], number of options, and responder [i.e., self-report or proxy]), number of items, whether the instrument was a preference-based measure (such that utility values can be generated directly for economic evaluation), and whether it was developed specifically for use in ALS. Extracted PROM data were checked collectively by 3 reviewers (E. McDool, J.C., P.A.P.).

**Table:** Inclusion and Exclusion Criteria

Inclusion	Exclusion
A: Paper inclusion criteria	
• Patients: Studies of adults (18+) with a diagnosis of ALS/MND • Intervention/exposure: Measures of health-related quality of life • Outcomes: Health-related quality of life • Studies: Qualitative and quantitative studies published as a full-text original article in English that include qualitative study data and/or use a multi-item PRO to assess HRQoL in people diagnosed with ALS/MND • Studies published since 2017	• Discussion articles or reviews without study data • Studies published in non-English language • Observational studies of etiology or onset • Studies that do not assess relevant outcomes or themes of interest that is not HRQoL within the patient • Studies that do not report HRQoL outcomes that are extractable for the ALS/MND sample • Studies published before 2017
B: PROM inclusion criteria	
• Multi-item PROs published in English assessing subjective HRQoL in pwALS/MND that did not require clinical input and for which all items were accessible by the study team to review	• PROMs requiring clinical input, information, or administration • PROMs that cannot be applied consistently across populations (i.e., adaptive measures) • Single item PROMs • Instruments without subjective assessment of HRQoL • Bespoke instruments or subsets of PROM items used • PROMs not published or translated to English

Abbreviations: ALS = amyotrophic lateral sclerosis; HRQoL = health-related quality of life; MND = motor neuron disease; PRO = patient-reported outcome; PROM = patient-reported outcome measure; pwALS = people with ALS.

An a priori framework, developed for a generic measure of health and well-being, the EQ Health and Wellbeing instrument (EQ-HWB),^17,18^ was used to inform the initial QuALS framework. The EQ-HWB framework was informed by the Wilson and Cleary model of HRQoL^19^ and a systematic review of qualitative literature.^17^ It includes 7 themes, including Activities; Physical sensations; Autonomy; Cognition; Feelings and emotions; Self-identity; and Relationships.^17^ The content of the items (i.e., questions) included within a PROM were reviewed, and themes extracted to refine the initial framework. A similar approach was taken with the qualitative data, where themes were extracted from quotations. Modifications were made iteratively to the QuALS framework during the data extraction process to ensure that the content more accurately depicted the content of the included PROMs and qualitative studies. Themes were extracted by 3 reviewers independently. Where disagreement occurred, HRQoL themes were ratified through group discussion.

After developing the initial draft QuALS framework, Patient and Public Involvement and Engagement (PPIE) feedback was obtained. The aim of the PPIE was to ratify and finalize the HRQoL framework based on the feedback from individuals with lived experience. PPIE was undertaken with 9 pwALS (and/or their informal carers) and 10 health care professionals (HCPs) through online workshops in September 2023. Attendees were asked to consider the relevance, comprehensiveness, and comprehensibility (i.e., understanding) of the developed framework and the accompanying description of the themes and subthemes within it.^20^ All comments and feedback from the PPIE workshops were recorded and considered before framework finalization. Details of the PPIE consultation process are provided (eAppendix 2). The content of included PROMs and qualitative studies was then mapped to the finalized QuALS framework by 2 researchers independently. If disagreement occurred, independent extraction was completed by a third reviewer. Remaining discrepancies were resolved through discussion by all 3 reviewers.

### Standard Protocol Approvals, Registrations, and Patient Consents

This study uses secondary data through a systematic review and thus institutional review board and patient consent are waived. Ethical approval is not required for collaborative patient involvement activities.

### Data Availability

Data supporting the findings of this study are contained in the manuscript and online supplement.

## Results

### Systematic Review

**Figure 1:**
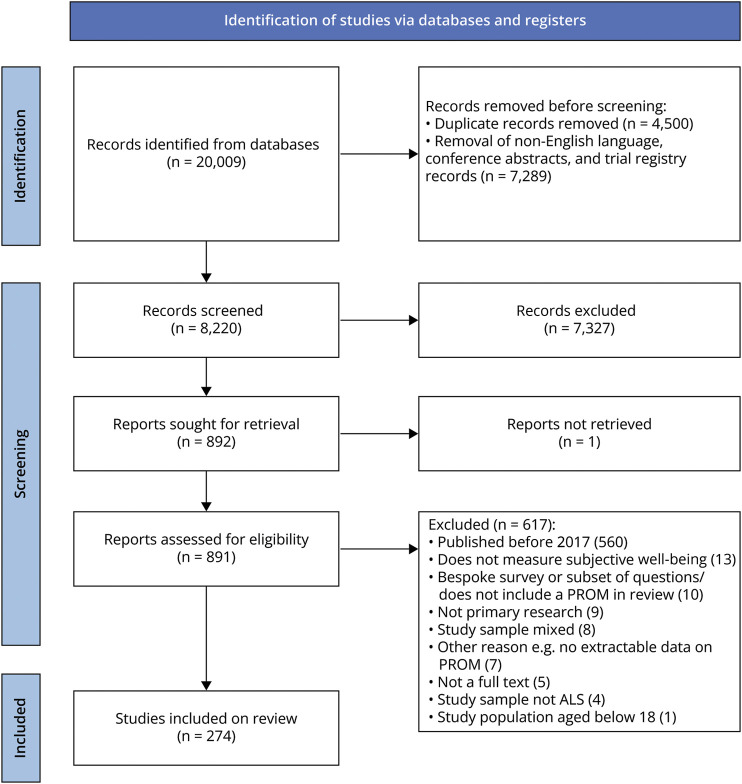
PRISMA Flow Diagram of Included Studies ALS = amyotrophic lateral sclerosis; PRISMA = Preferred Reporting Items for Systematic Reviews and Meta-Analyses; PROM = patient-reported outcome measure.

A total of 20,009 records were identified before the removal of duplicates and non-English texts, which, after removal, resulted in 8,220 records for screening (Figure 1). In total, 274 studies published during 2017–2022 were identified for inclusion (eAppendix 1, eTable 2). Eleven qualitative studies were included and, within these studies, 4 also used a PROM. Across all studies, 111 PROMs were administered to measure at least 1 aspect of HRQoL in ALS. On average, 2 PROMs were used per study, with a single PROM used in 115 studies and more than 2 PROMs used in 84 studies. In 3 separate studies, 7 PROMs were used^21-23^ (eAppendix 3). A full reference list of included studies is given in eAppendix 1.

The 111 PROMs used to assess (an aspect of) HRQoL in pwALS are detailed in eAppendix 1 (eTable 3). The most frequently used PROM was the ALS Functioning Rating Scale Revised (ALSFRS-R)^24^ (n = 224 citations), followed by the Hospital Anxiety and Depression Scale (HADS)^25^ (n = 29 citations) and the ALS Assessment Questionnaire-40 (ALSAQ-40)^26^ (n = 21 citations). Twenty-two PROMs developed specifically for use in pwALS were used. Generic HRQoL PROMs were also identified, with the most widely cited including the McGill Quality of Life Questionnaire^27^ (n = 12 citations) and the Short-Form-36 (SF-36)^28,29^ (n = 10 citations). Two PROMs were preference-based measures (EuroQol 5-Dimension 5 Level^30^ and EuroQol 5-Dimension 3-Level^31^), and utilities may be derived indirectly from a further 3 instruments (SF-12 version 2,^32^ SF-12 version 1,^33^ and SF-36^28,29^). The number of items in PROMs ranged from 3 (Patient-Reported Outcomes Measurement Information System pain intensity questionnaire^34^) to 93 (Quality of Life Enjoyment and Satisfaction Questionnaire^35^). Fifty-five PROMs had only 1 citation each (eAppendix 3).

### The QuALS Framework

**Figure 2:**
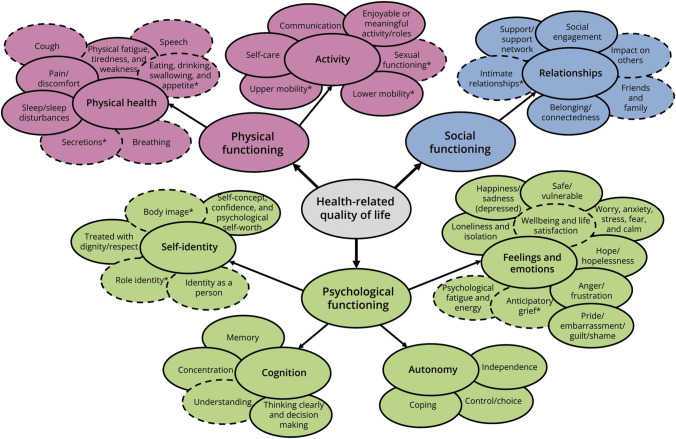
Comprehensive Conceptual Framework of Health-Related Quality of Life in Amyotrophic Lateral Sclerosis (QuALS) Ovals with a solid line indicate where the concept covered by a subtheme was originally included in the EQ Health and Wellbeing instrument (EQ-HWB) framework. This includes subthemes that have incurred minor changes (e.g., to the title to include additional aspects or reframe the concept). Ovals with a dashed line indicate a new concept for amyotrophic lateral sclerosis that was added as a result of extraction or Patient and Public Involvement and Engagement (PPIE) feedback. An asterisk indicates a subtheme that was amended specifically as a result of PPIE feedback.

The initial QuALS framework was inductively modified to reflect themes identified within the PROMs and/or qualitative quotations. Feedback from PPIE resulted in amendments being made to theme/subtheme titles and their descriptions, and location of subthemes within themes. Some subthemes were added (e.g., *intimate relationships*) or split (e.g., *mobility* split into *upper mobility* and *lower mobility*). All comments and resulting refinements are detailed in eAppendix 2. Figure 2 highlights the subthemes amended through PPIE.

The finalized QuALS framework (Figure 2) reflects the impact of ALS upon HRQoL categorized within the 3 HRQoL domains of physical, psychological, and social functioning.^2^ The QuALS framework includes 7 themes of HRQoL, covering Activities; Physical Health; Autonomy; Cognition; Feelings and Emotions; Self-identity; and Relationships. Across the 7 themes, 42 subthemes are covered, reflecting the broad range of HRQoL issues associated with ALS. Full descriptions of each subtheme are provided (eAppendix 1).

### Physical Functioning: Activity

This theme consists of 6 subthemes including *communication*, *sexual functioning*, *enjoyable or meaningful activities/roles*, *upper mobility*, *lower mobility*, and *self-care*. *Communication* was noted to include both verbal and nonverbal forms, and incorporates the use of communication devices, facial expressions, etc. The ability to have/maintain eye contact with others was particularly important for some pwALS. *Sexual functioning*, including interest in sex and satisfaction with sex life, was added as a subtheme after feedback from PPIE members. Limitations in everyday activities also feature the impact of ALS on the ability to do activities deemed to be enjoyable or meaningful (e.g., sports, work, hobbies). Relatedly, concepts relating to *self-care* were identified, including the ability to undertake activities relating to personal hygiene (e.g., toileting, washing).

*Mobility* was initially extracted as a single subtheme, however, after PPIE feedback, this was split into 2 separate subthemes covering *upper mobility* and *lower mobility* to address the distinct way that 1 or both can affect HRQoL. *Upper mobility* covers both gross and fine motor skills associated with movement in the upper body and hands, including but not limited to gripping objects, lifting, writing, and head and arm movement. *Lower mobility* also covers gross and fine motor skills, including, for example, walking, standing, and purposeful positioning of feet and toes.

### Physical Functioning: Physical Health

After feedback, the Physical Sensations theme was renamed Physical Health. It includes 8 subthemes: *Pain/discomfort*; *sleep/sleep disturbances*; *eating*, *drinking*, *swallowing*, *and appetite*; *cough*; *breathing*; *secretions*; *physical fatigue*, *tiredness*, *and weakness*; and *speech*. Pain was considered an important concept within the literature and PPIE and was combined with discomfort, because there is overlap in the way these are conceptualized and assessed in ALS (e.g., cramping, joint stiffness, tingling, and burning).

The *sleep/sleep disturbances* subtheme encompasses issues including getting to sleep and waking (e.g., nightmares) and sleeping at times where it would be preferable not to. This was considered distinct from *physical fatigue*, *tiredness*, *and weakness*, which covers physical fatigue, lack of energy, feeling weak (including muscle weakness), and related physical functioning concepts.

In PPIE, HCPs expressed that drinking should be added to the eating and appetite subtheme, which could also include swallowing, but not oral secretions. Consequently, the subtheme *eating*, *drinking*, *swallowing*, *and appetite* was considered more appropriately worded for pwALS. This theme covers difficulties with eating and loss of enjoyment in eating, in addition to changes in appetite, which may be accompanied by weight loss, and difficulties chewing and choking. Separately, *secretions* were included as a subtheme to reflect the changes in oral mucus and secretions experienced by pwALS based on HCP feedback.

*Breathing* and *cough* subthemes reflect breathing difficulties, aids and breathlessness, and cough and related difficulties, respectively. *Speech* was separated from *communication* to signify physical changes in the ability to speak.

### Psychological Functioning: Autonomy

This theme includes *coping*, *control/choice*, and *independence* subthemes. *Coping* encompasses concepts relating to the in/ability to cope (including with change), the maintenance of responsibilities, supporting others (e.g., with ALS, own friends/family), and acceptance or denial relating to the ALS diagnosis, treatment, and progression.

Another subtheme is *control/choice*, which denotes (lack of) control over choices to manage one's life, which may include treatment choices. Uncertainty, understanding, and awareness of choices available to pwALS, in addition to spontaneity and the ability to make unwise or risky decisions, are also included within this subtheme. PPIE attendees noted the importance of the *independence* subtheme, including pwALS's perceived independence and the ability to live independently.

### Psychological Functioning: Cognition

This theme consists of 4 subthemes, including *concentration*; *understanding*; *memory*; and *thinking clearly and decision making*. Some PPIE members noted that the Cognition theme may not affect everyone with ALS. Subsequently, an explanatory note was added to the QuALS framework (see eAppendix 1, eTable 4) to signal that the theme may be more prevalent in and relevant to some people (e.g., those living with frontotemporal dementia with ALS). It was also highlighted that many of the concepts within Cognition may be task dependent and this clarification was added to the subtheme description.

*Concentration*, including difficulty in concentrating and inattention, and the related concept of *memory*, including difficulties with both long-term and short-term memory and forgetfulness, were deemed relevant to pwALS. *Understanding* (of situations and conversations) features as a subtheme alongside *thinking clearly and decision making*, which includes confusion and the ability to make decisions. PPIE members noted that decreased flexibility and increased rigidity in decision making were related to this subtheme.

### Psychological Functioning: Feelings and Emotions

Ten subthemes feature within the Feelings and Emotions theme, including *happiness/sadness (depressed)*; *psychological fatigue and energy*; *hope/hopelessness*; *anticipatory grief*; *anger/frustration*; *worry*, *anxiety*, *stress*, *fear*, *and calm*; *safe/vulnerable*; *well-being and life satisfaction*; *pride/embarrassment/guilt/shame*; and *loneliness and isolation*.

*The happiness/sadness (depressed)* subtheme encompasses both positive and negative feelings of sadness, depression, suicidal thoughts, emotional pain, and crying episodes but also happiness, joy, and laughter (where genuine feelings are distinguished from the pseudobulbar affect, as highlighted by PPIE attendees). Relatedly, the *worry*, *anxiety*, *stress*, *fear*, *and calm* subtheme covers concepts of fear (e.g., of disease progression), distress, panic, but also calm. Related concepts including *anger and frustration* encompassing irritability, agitation, and violence were important and often associated with frustration with symptoms and situations.

*Hope/hopelessness* reflected the optimism or pessimism about the future experienced by pwALS, including feelings of helplessness. Alongside others, this subtheme was renamed after feedback from PPIE attendees that where possible, subthemes should be more positively or neutrally framed; hope, therefore, precedes hopelessness. A related but separate concept, which was noted as absent in the initial model by PPIE attendees, was *anticipatory grief* that may be experienced in mourning an expected future life.

Positive concepts of *wellbeing and life satisfaction* reflect enjoyment of life, life satisfaction, and enthusiasm (or the reversal of these feelings), alongside goal setting and achievement. Concepts and feelings of pride and accomplishment feature within a separate subtheme, which included a variety of self-conscious emotions relevant to pwALS, including guilt, shame, and embarrassment (e.g., around symptoms or effects of the disease).

Feelings of *safety and vulnerability* were endorsed by PPIE members, reflecting feelings of safety or lack of, including reliance upon others. The related concept of physical isolation (including feelings of being trapped or imprisoned) was highlighted by PPIE and subsequently included within *loneliness and isolation*, which includes feelings of psychological and emotional isolation or feeling un/welcome.

Although physical fatigue is included within physical health, the importance of *psychological fatigue and energy* was also apparent with motivation and apathy deemed important related concepts.

### Psychological Functioning: Self-Identity

The self-identity theme includes 5 subthemes including *role identity*; *identity as a person*; *treated with dignity/respect*; *self-concept, confidence, and psychological self-worth*; and *body image*.

*Role identity* specifically reflects the change in roles (e.g., parental, at work) that may occur and adapt over time and the associated sense of purpose in those roles. A distinct subtheme was extracted to reflect the individual's *identity as a person* beyond their illness that included being interacted with as such. Relatedly, being *treated with dignity and respect* was deemed a distinct and important concept by PPIE attendees, reflecting how individuals felt they were perceived or treated and includes experiences of stigma and discrimination (including by strangers, HCPs, etc).

*Self-concept*, *confidence*, *and psychological self-worth* also features in the framework covering concepts such as self-esteem, self-loathing, and worthlessness. Relatedly, *body image* was initially extracted and combined with this subtheme, but PPIE felt that it should be a distinct subtheme covering physical changes in appearance and comfort with one's body.

### Social Functioning: Relationships

Six subthemes relating to social and personal relationships are included in the Relationships theme, including *social engagement*; *support/support network*; *impact on others*; *friends and family*; *belonging/connectedness*; and *intimate relationships*. The *social engagement* subtheme reflects interest in other people or opportunities for participation in socializing for pwALS. Separately, feelings of being included/excluded are described by the *belonging and connectedness* subtheme, reflecting feelings associated with acceptance and being understood.

The *support/support network* subtheme includes feeling supported, for example, by friends, family, or professional support, who may also provide emotional support and comfort. Relatedly, *friends and family* describes the ability or inability to make and maintain friends and the impact of health on relationships and the dis/satisfaction with existing relationships. An associated subtheme, *impact on others*, reflects pwALS's perceptions of the impact on friends, family, or other close relationships. Physical and emotional intimacy is included as a distinct category describing the bond between partners; PPIE attendees highlighted that such relationships may change through the journey of living with ALS.

### Mapping PROMs to the QuALS Framework

**Figure 3:**
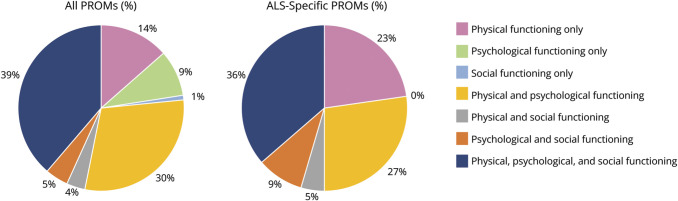
Proportion of PROMs Assessing the 3 High-Level Domains of HRQoL ALS = amyotrophic lateral sclerosis; HRQoL = health-related quality of life; PROM = patient-reported outcome measure.

Figure 3 indicates the proportion of PROMs and ALS-specific PROMs with content covering the 7 HRQoL themes. eFigure 2 summarizes the percentage of PROMs with content that covers the QuALS themes and subthemes (eAppendix 4 for full details of PROMs and their mapped content). Over a third of PROMs (n = 43, 39%) covered aspects of all 3 HRQoL domains (physical, psychological, and social functioning). Eight of these were ALS-specific PROMs. No single PROM assessed all identified subthemes within the QuALS framework. Across the 3 HRQoL domains, physical functioning was most commonly assessed with 86% (n = 95) of all PROMs, and 91% (n = 20) of ALS-specific PROMs assessing a minimum of 1 subtheme. Within ALS-specific PROMs, the proportion assessing physical functioning is higher and the proportion assessing psychological functioning is lower than in all PROMs overall.

The most frequently used instrument was the ALSFRS-R (N = 224 studies), which focused on physical functioning. The majority of studies using the ALSFRS-R (n = 130, 58%) used at least 1 other HRQoL PROM. The most commonly used instruments alongside the ALSFRS-R included the HADS (N = 22 studies), ALSAQ-40 (N = 16 studies), and the Epworth Sleepiness Scale (ESS)^36^ (N = 9 studies). The use of the ALSFRS-R combined with HADS alone does not cover social functioning. Even in studies wherein as many as 6 additional PROMs were used alongside the ALSFRS-R, not all subthemes within the QuALS framework were covered, though aspects of each of the 3 high-level HRQoL domains were assessed.^22,23^

Despite physical functioning being the most prevalent high-level HRQoL domain, no single PROM contained item content that covered all physical subthemes. Subthemes within the Feelings and Emotions theme were most commonly assessed across all PROMs including *happiness/sadness (depression)* (n = 54, 49% of all PROMs), *worry*, *anxiety*, *stress*, *fear*, *and calm* (n = 48, 43%), and *psychological fatigue* (n = 44, 40%). In MND-specific PROMs, commonly assessed themes included *speech* (n = 14, 64%); *lower mobility* (n = 13, 59%); and *eating*, *drinking*, *swallowing*, *and appetite* (n = 13, 59%). Some subthemes deemed important by PPIE members were assessed by very few, or no PROMs, for example: *treated with dignity/respect* (assessed in only 6% of all PROMs), *role identity* (5%), *safe/vulnerable* (4%), and *anticipatory grief* (0%).

Qualitative studies presented more adequate coverage of all 3 high-level HRQoL domains (i.e., physical, psychological, and social functioning) with 9 studies (81%) containing quotations relating to all domains. The remaining studies did not feature content about social functioning. The most common subtheme assessed and extracted from the qualitative data was *speech* (within Physical Health) (n = 8, 73%).

## Discussion

This study presents a newly developed conceptual framework of HRQoL in ALS (QuALS) based on a comprehensive systematic review and stakeholder engagement. Evidence from both quantitative and qualitative assessments of the impacts of ALS on HRQoL informed the framework. QuALS was ratified and approved by people with lived experience and experts in ALS. The QuALS framework is available for use to guide future research and outcomes measurement on HRQoL in pwALS.

The QuALS framework includes 42 subthemes of HRQoL, indicating the breadth of issues that are potentially relevant to pwALS. The mapping exercise (eAppendix 4) highlighted that no single qualitative study or PROM captured all aspects of HRQoL within the QuALS framework and only 39% of all PROMs covered at least 1 subtheme from each of the high-level HRQoL domains: physical, psychological, and social functioning. Furthermore, some subthemes deemed important by PPIE members were assessed by no, or very few PROMs, thereby revealing gaps in existing research. As the comprehensiveness of existing measures judged against the QuALS framework is limited, it may be that the extent to which ALS affects people's HRQoL is not fully recognized or quantified.

Many PROMs developed for use in ALS, including the most used measure, the ALSFRS-R, only assess aspects of physical functioning. Even within this high-level domain, ALS-specific PROMs did not include content covering all subthemes identified in the QuALS framework. The ALSFRS-R was developed for the evaluation of functional status and functional change in patients.^37^ The instrument correlates highly with clinical progression^24^ and may, therefore, be used in some studies to measure ALS stage.^9^ When applying the QuALS framework to ALSFRS-R, notable aspects of physical functioning were not reflected under both the Activity and Physical Health themes, including *pain/discomfort*, *sleep/sleep disturbances*, *physical fatigue*, *tiredness and weakness*, *sexual functioning*, and *enjoyable or meaningful activities/roles*. Accordingly, using the ALSFRS-R as a primary or exclusive outcome measure potentially misses important aspects of HRQoL relevant to pwALS (including psychological and/or social impacts). Some studies use supplementary instruments (e.g., HADS, ESS) alongside the ALSFRS-R to improve coverage or assess particular aspects of interest; however, even in these cases, broad HRQoL themes continue to be missed. Future work exploring the measurement of HRQoL in ALS can use the QuALS to ensure aspects identified as important to pwALS are considered for assessment.

The hierarchical structuring of subthemes within the QuALS framework was informed by PPIE feedback and emphasizes the subjective impact of ALS on different aspects of HRQoL. It could be argued that some themes or subthemes, such as cognition (psychological functioning), could also be situated within an alternative domain, such as physical functioning. However, it is assumed that the subjective impact on pwALS is the same. QuALS can be iteratively refined and/or extended based on further research, including work to establish the relative importance of different HRQoL subthemes to pwALS. Although frequency and coverage of thematic content in PROMs may reflect research interest, it does not necessarily define what is important to pwALS.

Our review adopted a rigorous approach to screening, data extraction, and the classification of HRQoL themes, using dual coding and reviewer triangulation to enhance review quality.^38^ This was complemented by input from people with a range of lived experiences and HCPs to ratify and confirm the QuALS framework. The content of all PROMs identified was mapped to a comprehensive matrix of aspects of HRQoL to aid PROM selection based on content coverage and is available for use as a supplementary resource (eAppendix 4).

Although every effort was made to ensure diversity of individuals providing PPIE input, additional characteristics could have been considered, including employment status and parental status. Furthermore, as the PPIE opportunity was restricted to individuals living in the United Kingdom, the QuALS framework was verified within the UK context. Future research may wish to incorporate the views of pwALS and HCPs outside of the United Kingdom. Nevertheless, the studies included in the review were not restricted by geographical location; therefore, the review itself was international in scope.

Owing to the size and scope of this review, articles published before 2017 were not included and this may have led to additional PROMs being omitted. However, it can be argued that our approach led to a focus on PROMs (and thus HRQoL themes) used within contemporary literature. Although it is plausible that unidentified legacy PROMs may contain items that differ in content to the identified PROMs, this is unlikely to alter the comprehensiveness of the QuALS framework, which was verified by PPIE stakeholders. Finally, it should be noted that the themes and subthemes of PROMs and qualitative studies included in this review may differ from the scoring system or thematic analysis proposed by the developers.

The novel QuALS framework highlights the breadth of HRQoL issues relevant to pwALS, featuring 42 subthemes. This comprehensive framework was developed through a robust review of the existing literature, supplemented by PPIE consultation with key stakeholders including people with lived experience. Our research highlights clear gaps in aspects of HRQoL that have not been adequately assessed in prior work. Although most outcome measures in ALS have focused on physical functioning, no single PROM covered all physical functioning subthemes included in the QuALS framework. In measuring ALS outcomes, there is, therefore, a risk of missing important aspects of physical, psychological, and social functioning that matter to pwALS.

The QuALS framework is a useful and informative resource for researchers, clinicians, and stakeholders who are interested in better understanding and assessing the impact of ALS on HRQoL. QuALS enables a holistic approach to measuring HRQoL and can be used as a basis for PROM selection, development, or evaluation. Furthermore, QuALS may aid care management decisions by providing a framework on which all relevant HRQoL outcomes for pwALS could be considered, including when evaluating new treatments and therapies.

## Appendix Authors

**Table:**

Name	Location	Contribution
Emily McDool, PhD	University of Sheffield, United Kingdom	Drafting/revision of the manuscript for content; major role in the acquisition of data; study concept or design; analysis or interpretation of data
Jill Carlton, PhD	University of Sheffield, United Kingdom	Drafting/revision of the manuscript for content; major role in the acquisition of data; study concept or design; analysis or interpretation of data
Philip A. Powell, PhD	University of Sheffield, United Kingdom	Drafting/revision of the manuscript for content; major role in the acquisition of data; study concept or design; analysis or interpretation of data
Elizabeth Coates, PhD	University of Sheffield, United Kingdom	Drafting/revision of the manuscript for content; major role in the acquisition of data
Liam Knox, PhD	University of Sheffield, United Kingdom	Drafting/revision of the manuscript for content; major role in the acquisition of data
Emily Mayberry, PhD, DClinPsy	University of Sheffield, United Kingdom	Drafting/revision of the manuscript for content; major role in the acquisition of data
Niamh Appleby	University of Sheffield, United Kingdom	Major role in the acquisition of data
Alys W. Griffiths, PhD	University of Sheffield, United Kingdom	Drafting/revision of the manuscript for content
Esther Hobson, PhD	University of Sheffield, United Kingdom	Drafting/revision of the manuscript for content; analysis or interpretation of data
Christopher J. McDermott, PhD	University of Sheffield, United Kingdom	Drafting/revision of the manuscript for content; study concept or design; analysis or interpretation of data

## Glossary

ALS: amyotrophic lateral sclerosis
ALSAQ-40: ALS Assessment Questionnaire-40
ALSFRS-R: ALS Functioning Rating Scale Revised
EQ-HWB: EQ Health and Wellbeing instrument
ESS: Epworth Sleepiness Scale
HADS: Hospital Anxiety and Depression Scale
HCP: health care professional
HRQoL: health-related quality of life
MND: motor neuron disease
PPIE: Patient and Public Involvement and Engagement
PROM: patient-reported outcome measure
pwALS: people with ALS
QuALS: Health-related Quality of life in Amyotrophic Lateral Sclerosis
SF-36: Short-Form-36
